# IMPROVING ACCESS TO DEMOGRAPHIC DATA RESOURCES FOR THE AGING NETWORK IN VIRGINIA

**DOI:** 10.1093/geroni/igad104.1904

**Published:** 2023-12-21

**Authors:** Sol Baik, Qian Cai, Tolu Odukoya

**Affiliations:** University of Virginia, Charlottesville, Virginia, United States; University of Virginia, Charlottesville, Virginia, United States; University of Virginia, Charlottesville, Virginia, United States

## Abstract

The statewide aging networks and their partner organizations are committed to data-driven communication and advocacy strategies for the needs of the aging population and betterment of their lives. High quality demographic data helps the professionals in the aging network to thoughtfully plan the allocation of resources and carefully target service delivery to those with greater needs. To promote national, state, and local initiatives, improving access to credible and timely data about aging population is crucial. In this presentation, we will describe the efforts to make data available and accessible to the aging network by offering an example of the demographic data services for the Area Agencies on Aging and affiliated organizations in Virginia. We will also share trials and errors to provide the data in a practical, streamlined, and user-friendly way. Finally, we will discuss the strategies and challenges in building effective and sustainable partnerships with a variety of state stakeholders.

